# Maternal mortality in the Middle East and North Africa region – how could countries move towards obstetric transition stage 5?

**DOI:** 10.1186/s12884-022-04886-7

**Published:** 2022-07-08

**Authors:** Sathyanarayanan Doraiswamy, Sohaila Cheema, Patrick Maisonneuve, Anupama Jithesh, Ravinder Mamtani

**Affiliations:** 1 Institute for Population Health, Weill Cornell Medicinem Education City, Qatar Foundation, Doha, Qatar P.O. Box 24144,; 2 IEO, European Institute of Oncology IRCCS, Milan, Italy

**Keywords:** Maternal mortality, Middle East and North Africa, Three delays, Obstetric transition stages

## Abstract

**Background:**

Maternal mortality in the Middle East and North Africa (MENA) region decreased significantly between 1990 and 2017. This was uneven, however, with some countries faring much better than others.

**Methods:**

We undertook a trend analysis of Maternal Mortality Ratios (MMRs) of countries in the region in order to understand differences in reduction across countries. Data were extracted from several databases for 23 countries and territories in the region on measures of women’s empowerment, availability of vehicles and human resources for health (as a proxy to the three delays model). We identified factors associated with MMR by grouping countries into five different Stages (I-V) of obstetric transition from high to low MMRs.

**Results:**

Among the four Stage II countries, MMR is associated with “antenatal care coverage (% with at least one visit)” and “medical doctors per 10,000 population”. Among the eight Stage III countries, MMR is associated with “Gender Parity Index in primary and secondary level school enrolment” and with “nursing and midwifery personnel per 10,000 population”. Among the 10 countries and one territory in Stages IV and V, MMR is associated with “GDP per capita”, “nursing and midwifery personnel”, and “motor vehicle ownership/motorization rate”. Two factors were associated with changes in MMR from the period 2006–2010 to 2011–2015: 1) change in adolescent birth rate (*r =* 0.90, *p* = 0.005) and 2) Gender Parity Index in primary level school enrolment (*r =* − 0.51, *p* = 0.04).

**Conclusion:**

Though impressive reductions in MMR have been achieved across countries in the MENA region since 1990, governments should realize that there exists an opportunity to learn from each other to bring MMRs as close to zero as possible. Immediate steps in the right direction would include investment in human resources for health, particularly nurses and midwives; measures to improve adolescent sexual and reproductive health; and greater investments in achieving gender equity in education.

**Supplementary Information:**

The online version contains supplementary material available at 10.1186/s12884-022-04886-7.

## Background

Maternal mortality is defined “as death that occurs to a woman as a direct result of obstetric complications or indirectly as a result of pregnancy-induced exacerbation of pre-existing medical conditions, but not as a result of incidental or accidental causes” [1]. Maternal mortality is generally considered a good indicator of a country’s health system performance and at the same time, socio-economic theories have helped to explain reduction of maternal mortality among populations that have advanced in wealth and education [2, 3]. The wealth-education-health triad is a well-known social and structural determinant of health. Maternal mortality ratios (MMRs) decreased by 38.4% between 1990 and 2017 globally, whereas it declined by 46.6% in the Northern Africa and Western Asia region (a proxy for the Middle East and North Africa region, (MENA)) [4]. In 2017, the global point estimate of MMR was 211/100,000 live births and for Northern Africa and Western Asia 81/100,000 live births [4]. MMRs decrease in MENA countries is, however, unequal. Although some countries have made remarkable progress over the past two decades, improvement in further reduction of MMR slowed significantly in recent years [5]. The progressive decrease of MMR in some MENA countries in comparison to others provides an opportunity for countries that are currently lagging behind to analyze factors for success and lessons learned in other MENA countries.

Since MENA countries have varying MMRs, a trend analysis of MMRs in these countries during the last two decades may be beneficial. Souza et al. grouped countries according to their MMRs in five stages (Stage I: MMR > 1000/100,000 live births; Stage II: 300–999; Stage III: 50 - < 299; Stage IV: < 50; and Stage V: Very low MMR (accepted to be < 5) [6]. They also reported various country-level policy interventions likely to yield optimal outcomes during the five stages.

Programmatically speaking, the three delays model of Thaddeus and Maine (1994) is considered a useful framework to design high impact interventions. This model considers these three delays as explaining the reasoning behind maternal deaths: a) delay in seeking care (lack of empowerment of women) – *first delay*; b) delay in reaching facilities (failure of transport and communications) – *second delay*; and c) delay in provision of care (deficiencies in human resource and commodity availability) – *third delay* [7]. There is a known relationship between the delays found in a country and that country’s obstetric stage. A preponderance of first and second delays is predicted for Stage I and II countries, the third delay is more commonly found in Stage IV and V countries, and there is equal contribution from all three delays in Stage III countries [6]. Our objective is to study factors (linked to the three delays model) associated with reductions in MMRs in MENA countries according to their respective obstetric stages.

## Methods

MMRs were retrieved for the period 1990–2015 from the official United Nations website for the Millennium Development Goals (MDG) indicators for 23 countries/territories (22 countries + one territory) in the MENA region [8]. We defined MENA region using the definition from the United Nations Children’s Fund for the MENA region and also included countries from the Eastern Mediterranean Region of the World Health Organization (WHO) for comprehensiveness [9, 10]. These together included Afghanistan, Algeria, Bahrain, Djibouti, Egypt, Islamic Republic of Iran, Iraq, Jordan, Kuwait, Lebanon, Libyan Arab Jamahiriya, Morocco, Oman, Pakistan, Qatar, Saudi Arabia, Somalia, State of Palestine Sudan, Syrian Arab Republic, Tunisia, United Arab Emirates and Yemen. Countries are grouped according to the obstetric stages (I to V), based on their 2015 MMRs [6, 10].

Using the three delays framework, we extracted data for the 23 countries/territories on the measures of women’s empowerment, availability of vehicles and human resources for health. In addition, we also obtained data on health system performance related to reproductive health care and overall wealth of the country. These data were extracted from the United Nations report on the Millennium Development Goals for women’s empowerment and health system performance, WHO’s global observatory data on human resources for health, World Bank data on gross domestic product, International Organization of Motor Vehicle Manufacturers and the Our World in Data project of the global change data lab for transport availability [8, 10–14].

We calculated national average MMRs for the period 2011 to 2015 (when missing for 2015 annual data from 2016 and 2017 were taken) and looked for associations with different variables/ indicators (expressed in percentages). We omitted variables represented by raw numbers. Correlation between the various indicators and MMR was represented by scatterplots. To improve normality of the distribution, MMRs and GDPs were log-transformed.

## Results

**Fig. 1:**
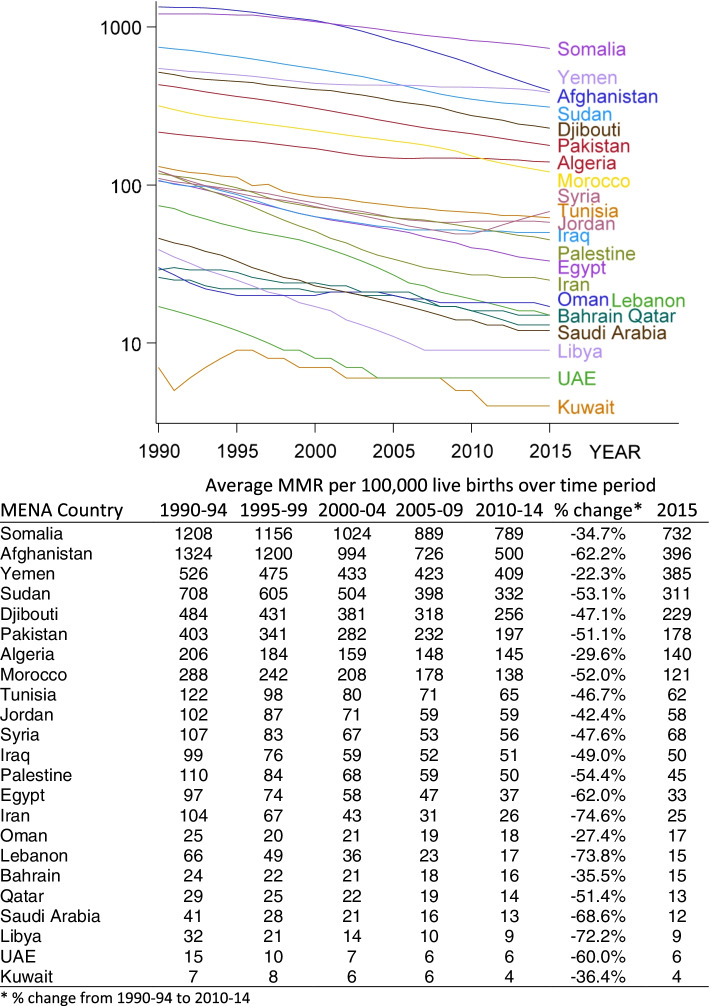
Change in MMR over time in MENA countries

MMR demonstrates a decreasing trend over the last 25 years in all MENA countries. The relative reduction from the period 1990–1994 to the period 2010–2014 ranged from 22.3% in Yemen to 74.6% in Iran. In 2015, the highest MMRs were reported in Somalia (732), Afghanistan (396), Yemen (385) and Sudan (311), while the lowest MMRs were reported in Kuwait [4], the United Arab Emirates [6] and Libya [9]. Change over time is shown in Fig. 1 below. While all countries have seen a further reduction of their 2010–14 maternal mortality levels in 2015, Syria stands out an exception with the MMR climbing back to almost its levels in 2000–2004 –an effect of the protracted conflict.

GDP per capita (US$) is correlated with MMR and many of the selected indicators (Supplementary Table 1). Table 1 presents association between selected indicators and respective obstetric stage (average values reported during 2011–2015) of countries. A particularly strong inverse correlation is observed between GDP and MMR (*r =* − 0.90), while strong positive correlations are observed with “nursing and midwifery personnel per 10,000 population” (*r =* 0.83), “medical doctors per 10,000 population” (*r =* 0.72) and “proportion of births attended by skilled health personnel” (*r =* 0.69). As a measure of wealth, GDP is also strongly correlated with “motor vehicle ownership” (*r =* 0.85) and “motorization rate” (*r =* 0.75) [15, 16].

**Table 1:** Association between selected indicators and obstetric stage (average values reported during 2011–2015)

	# of countries with available data	Stages IV&V (MMR < 5 &5–50)	Stage III (MMR 50–299)	Stage II (MMR 300–999)	Test for linear trend
		Mean ± SD	Mean ± SD	Mean ± SD	*p-*value
MENA COUNTRIES, n	23	11	8	4	
GDP per capita, US$	23	**23,773 ± 23,588**	**3515 ± 1746**	**1005 ± 651**	**< 0.0001**
Proportion of births attended by skilled health personnel, %	13	**97 ± 3.8**	**85 ± 18.9**	**42 ± 4.2**	**0.002**
Contraceptive use among married women 15–49 years, any method, %	12	57.7 ± 19.5	50.3 ± 17.2	27.5 ± 9.2	0.09
Contraceptive use among married women 15–49 years, modern methods, %	12	49.3 ± 13.3	39.0 ± 14.1	22.5 ± 3.5	0.05
Adolescent birth rate, per 1000 women	9	23.3 ± 18.3	9.2 ± 3.4	67.0	0.05
Antenatal care coverage (At least one visit), %	14	**94.3 ± 4.4**	**86.6 ± 10.6**	**62.3 ± 15.6**	**0.002**
Antenatal care coverage (At least four visits), %	12	**87.7 ± 7.2**	**58.7 ± 25.3**	**33.0 ± 25.5**	**0.03**
Unmet need for family planning, total, %	10	**10.3 ± 3.8**	**11.3 ± 4.6**	**29.0**	**0.008**
Unmet need for family planning, spacing, %	10	**5.0 ± 1.7**	**5.3 ± 2.0**	**15.0**	**0.003**
Unmet need for family planning, limiting, %	10	**5.0 ± 3.0**	**6.2 ± 2.9**	**14.0**	**0.03**
Gender Parity Index in primary school enrolment, girls/boys ratio	17	**0.99 ± 0.05**	**0.94 ± 0.04**	**0.81 ± 0.09**	**0.0004**
Gender Parity Index in secondary school enrolment, girls/boys ratio	18	**1.01 ± 0.07**	**0.93 ± 0.13**	**0.71 ± 0.19**	**0.002**
Gender Parity Index in tertiary level school enrolment, girls/boys ratio	18	1.96 ± 1.75	1.15 ± 0.35	0.62 ± 0.42	0.15
Share of women in wage employment in the non-agricultural sector, %	9	16.5 ± 3.5	20.6 ± 4.8	–	0.18
Proportion of seats held by women in national parliament, %	22	7.1 ± 6.1	18.8 ± 6.4	15.9 ± 12.6	0.07
Nursing and midwifery personnel, per 10,000 population	22	**41.5 ± 20.2**	**16.7 ± 9.3**	**5.3 ± 0.6**	**0.0007**
Medical doctors, per 10,000 population	22	**18.0 ± 6.3**	**11.7 ± 6.4**	**2.6 ± 1.6**	**0.0003**
Motor vehicle ownership, per 1000 population	23	**315 ± 181**	**80 ± 51**	**23 ± 14**	**0.001**
Motorization rate 2014–15, per 1000 population	21	**259 ± 155**	**142 ± 104**	**29 ± 25**	**0.01**

The countries organized into the four stages of obstetric transition based on their 2015 levels were: 1) one country (Kuwait) has reached stage V (with MMR < 5 per 100,000 live births) 2) nine countries and one territory (UAE, Libya, Saudi Arabia, Qatar, Bahrain, Lebanon, Oman, Iran, Egypt and Palestine) were categorized as Stage IV (with MMR 5–50 per 100,000 live births) 3) eight countries (Iraq, Jordan, Syria, Tunisia, Morocco, Algeria, Pakistan, and Djibouti) were categorized as Stage III (with MMR of 50–300/100,000); and 4) four countries (Sudan, Yemen, Afghanistan and Somalia) were categorized as Stage II (with MMR of 300–1000/100,000). No country within the MENA region had an MMR > 1000/100,000 (Stage I). Since there was only one country in stage V (Kuwait), we combined it with others in stage IV for analysis purposes.

Significant differences between the three groups of countries (stages II, III, IV&V) were observed for most indicators during the most recent period (2011–2015) (Table 2). Average GDP per capita decreased from $23,773 (Stages-IV&V) to $3515 (Stage-III) and $1005 (Stage-II). Notably, large differences in the number of nursing and midwifery personnel per 10,000 population (41.5, Stages-IV&V; 16.7, Stage-III and 5.3, Stage-II) and medical doctors per 10,000 population (18.0, Stages-IV&V, 11.7, Stage-III and 2.6, Stage-II) were also observed (Table 1).

**Table 2:** Association between selected indicators and MMR based on average values reported during 2011–2015, using linear regression

	Pearson Correlation Coefficient	Univariable Linear regression	Multivariable linear regression adjusted for “GDP per capita”	Multivariable linear regression adjusted for “Nursing & midwifery personnel”
	R	Mean Square (*p-*value)	Adjusted Mean Square (*p-*value)	Adjusted Mean Square (*p-*value)
GDP (US$)^a^	**−0.90**	**39.5 (<.0001)**	–	**6.53 (0.0005)**
Proportion of births attended by skilled health personnel	**−0.79**	**8.57 (0.001)**	0.23 (0.46)	0.83 (0.29)
Contraceptive use among married women 15–49 years old, any method	**−0.58**	**4.59 (0.046)**	1.04 (0.09)	2.17 (0.08)
Contraceptive use among married women 15–49 years old, modern methods	−0.57	4.45 (0.051)	0.88 (0.13)	1.99 (0.09)
Adolescent birth rate	0.55	4.50 (0.13)	0.17 (0.53)	0.04 (0.82)
Antenatal care coverage (At least one visit)	**−0.71**	**7.80 (0.004)**	0.76 (0.15)	1.26 (0.18)
Antenatal care coverage (At least four visits)	**−0.79**	**7.27 (0.002)**	0.55 (0.24)	0.26 (0.55)
Unmet need for family planning, total	**0.68**	**4.23 (0.03)**	0.41 (0.33)	1.15 (0.22)
Unmet need for family planning, spacing	0.60	3.36 (0.07)	0.92 (0.14)	1.65 (0.13)
Unmet need for family planning, limiting	**0.69**	**4.42 (0.03)**	0.07 (0.68)	0.61 (0.37)
Gender Parity Index in primary school enrolment	**−0.73**	**14.4 (0.0009)**	0.79 (0.19)	1.56 (0.14)
Gender Parity Index in secondary school enrolment	**−0.72**	**13.5 (0.0002)**	0.42 (0.32)	0.88 (0.28)
Gender Parity Index in tertiary level school enrolment	**−0.48**	**7.36 (0.046)**	**1.64 (0.05)**	0.41 (0.47)
Share of women in wage employment in the non-agricultural sector	0.44	1.34 (0.24)	0.17 (0.54)	0.42 (0.46)
Proportion of seats held by women in national parliament	0.39	7.20 (0.08)	0.07 (0.69)	0.64 (0.35)
Nursing and midwifery personnel	**−0.85**	**34.7 (<.0001)**	1.36 (0.07)	–
Medical doctors	**−0.81**	**31.5 (<.0001)**	**2.34 (0.01)**	2.35 (0.06)
Motor vehicle ownership	**−0.81**	**31.9 (<.0001)**	0.28 (0.43)	**3.59 (0.02)**
Motorization rate 2014–15	**−0.74**	**21.0 (0.0001)**	0.76 (0.19)	0.83 (0.27)

^a^ Log-transformed values

Mean squares and p-value are obtained from univariable and multivariable linear regression models adjusted for GDP or Nursing and midwifery personnel

At univariate analysis, significant correlations were observed between MMR (log-transformed to improve normality) and most of the selected indicators (Table 2). The strongest correlation was found between MMR and “GDP per capita” (*r =* − 0.90) and between MMR and “nursing and midwifery personnel” (*r =* − 0.85). After single adjustment for GDP, only “Gender Parity Index in tertiary level enrolment” (*p* = 0.05) and “number of medical doctors per 10,000 population” (*p* = 0.01) remain associated with MMR, but the magnitude of the association reduced considerably. Alternatively, after adjustment for “nursing and midwifery personnel”, GDP and to a lesser extent “motor vehicle ownership”, which itself is correlated with GDP, remain independent predictors of MMR.

Among the four Stage II countries, MMR was associated with “antenatal care coverage (% with at least one visit)” and “medical doctors per 10,000 population”. Among the eight Stage III countries, MMR is associated with “Gender Parity Index in primary and secondary level enrolment” and with “nursing and midwifery personnel per 10,000 population”. Among the 11 Stage IV&V countries, MMR is associated with “GDP per capita”, “nursing and midwifery personnel” and “motor vehicle ownership /motorization rate” (Table 3).

**Table 3:** Association between selected indicators and MMR based on average values reported during 2011–2015 in countries in different stages

	Stage II Countries (***n =*** 4)	Stage III Countries (***n =*** 8)	Stages IV&V Countries (***n =*** 11)
	Correlation coefficient (*P-*value)	Correlation coefficient (*P-*value)	Correlation coefficient (*P-*value)
GDP (US$) ^a^	**−0.95 (0.05)**	−0.65 (0.08)	**− 0.74 (0.008)**
Proportion of births attended by skilled health personnel	–	−0.55 (0.20)	− 0.32 (0.68)
Contraceptive use among married women 15–49 years, any method	–	− 0.68 (0.09)	0.68 (0.52)
Contraceptive use among married women 15–49 years, modern methods	–	− 0.42 (0.35)	0.94 (0.20)
Adolescent birth rate	–	–	0.76 (0.08)
Antenatal care coverage (At least one visit)	**−0.99 (0.008)**	−0.35 (0.45)	0.57 (0.43)
Antenatal care coverage (At least four visits)	0.68	−0.72 (0.07)	0.59 (0.60)
Unmet need for family planning, total		0.71 (0.11)	−0.17 (0.89)
Unmet need for family planning, spacing		0.69 (0.13)	−0.85 (0.36)
Unmet need for family planning, limiting		0.69 (0.13)	0.32 (0.79)
Gender Parity Index in primary school enrolment	−0.95 (0.20)	**−0.89 (0.007)**	0.11 (0.81)
Gender Parity Index in secondary school enrolment	−0.99 (0.07)	**− 0.80 (0.03)**	0.20 (0.64)
Gender Parity Index in tertiary level school enrolment	−0.96 (0.17)	− 0.51 (0.31)	− 0.29 (0.45)
Share of women in wage employment in the non-agricultural sector	–	− 0.21 (0.79)	0.36 (0.55)
Proportion of seats held by women in national parliament	−0.28 (0.72)	− 0.15 (0.72)	− 0.49 (0.15)
Nursing and midwifery personnel	− 0.93 (0.06)	**− 0.76 (0.03)**	**−0.72 (0.02)**
Medical doctors	**−0.99 (0.003)**	−0.50 (0.20)	− 0.63 (0.05)
Motor vehicle ownership	−0.86 (0.14)	− 0.54 (0.16)	**−0.68 (0.02)**
Motorization rate 2014–15	0.99 (0.09)	−0.52 (0.23)	**−0.70 (0.02)**

^a^ Log-transformed values

**Fig. 2:**
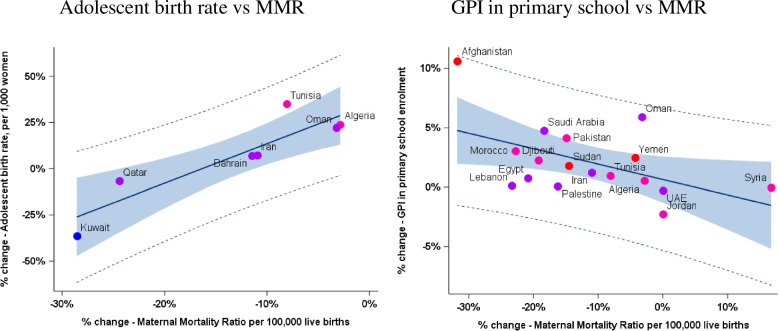
Correlation between change in selected indicators and change in maternal mortality (average change from 2006 to 2010 to 2011–2015) GPI: Gender parity index; Red dots indicate Stage II countries, purple dots Stage III countries, and blue dots Stages IV and V countries

We then assessed whether recent changes (from 2006 to 2010 to 2011–2015) of the selected indicators could be associated with parallel changes in MMR (Table 4). Only two factors were associated with MMR reduction: “Change in adolescent birth rate” (*r =* 0.90, *p* = 0.005) and “Gender Parity Index in primary level school enrolment” (*r =* − 0.51, *p* = 0.04). Correlations for these two factors are displayed in Fig. 2.

**Table 4:** Association between changes in selected indicators and changes in maternal mortality (average change from 2006 to 2010 to 2011–2015)

Change in indicator	Change in Maternal Mortality Ratio (MMR) per 100,000 live births	
	Number of countries with	Pearson Correlation
	**available data**	**Coefficient (*p-*value)**
GDP (US$) *	22	−0.24 (0.27)
Proportion of births attended by skilled health personnel	11	−0.29 (0.38)
Contraceptive use among married women 15–49 years, any method	9	0.07 (0.86)
Contraceptive use among married women 15–49 years, modern methods	9	− 0.34 (0.37)
Adolescent birth rate	7	**0.90 (0.005)**
Antenatal care coverage (At least one visit)	12	0.11 (0.74)
Antenatal care coverage (At least four visits)	7	0.24 (0.61)
Unmet need for family planning, total	4	−0.02 (0.98)
Unmet need for family planning, spacing	4	−0.47 (0.53)
Unmet need for family planning, limiting	4	0.25 (0.75)
Gender Parity Index in primary school enrolment	17	**−0.51 (0.04)**
Gender Parity Index in secondary school enrolment	17	−0.20 (0.44)
Gender Parity Index in tertiary level school enrolment	16	−0.21 (0.43)
Share of women in wage employment in the non-agricultural sector	8	0.26 (0.53)
Proportion of seats held by women in national parliament	19	0.00 (0.99)
Nursing and midwifery personnel	20	0.14 (0.56)
Medical doctors	21	0.02 (0.92)
Motor vehicle ownership	21	−0.14 (0.54)

## Discussion

GDP per capita remains a decisive factor in determining the odds of a woman surviving pregnancy or childbirth in the MENA region, indicating prevailing inequality in the world [17]. In 2015, Somalia, Afghanistan, Yemen, and Sudan still had MMRs above 300 per 100,000 live births. These countries, however, apart from being affected by low GDPs, have also been negatively influenced by widespread conflict over several years [18]. Syria (stage III) requires special mention as the only country in the region in which the MMR has continued to increase during the period 2010–15, coinciding with the period of high intensity conflict [18]. Among the wealthier MENA countries in Stages IV and V, reductions of MMR continued with increasing wealth of the country [19]. A 1 % increase in GDP has been shown to produce 0.5% reduction in MMR [20]. The wealthier MENA countries enjoy better status in terms of women empowerment, transport, communication and stronger health systems. In addition to the vast gap in wealth level, we found a higher number of nursing and midwifery personnel and medical doctors in countries with the lowest MMRs. This is a significant finding, from which poor countries can learn an important lesson—the need for continued strategic investment in retaining and recruiting personnel for maternal health. The value of investing in human resources for maternal health in low-to-middle income countries has been emphasized by many experts [21, 22]. Continued investments in education to harness human capital are an important precursor to investing in human resources for health.

Among countries in Stages III-V, the number of nursing and midwifery personnel had a major impact on MMRs, while medical doctors played a more prominent role in Stage II countries. This is consistent with other findings from peer-reviewed literature. Midwives, when well supported, can provide 87% of reproductive health care including basic emergency obstetric and neonatal care according to the state of the world midwifery report in 2014 [23]. Medical doctors, on the other hand, play a critical role in emergencies, which cannot be managed by midwives in peripheral locations [24]. Our findings and observations made by others reflect inadequate support provided to midwives and nurses in Stage II countries, who struggle to make a difference in peripheral facilities [25–27]. Nurses and midwives in Stages III-V countries are likely to have better support systems to enable them to perform their duties at a higher level, being supported by medical doctors when complications occur. These findings emphasize urgent needs for continued investment in human resources for health.

What is novel in our analysis is that we found a strong association between the Gender Parity Index in primary and secondary school enrollment of girls and MMR in Stage III countries. This finding is supported by other studies [24]. Our analysis did not reveal any association between woman education and MMR in Stage II and Stages IV-V countries, presumably due to the strong influence of GDP per capita in these countries. Though weakly correlated, we found that gender parity in primary school enrollment was one of the two factors that could explain the change in maternal mortality over time in the region. Stage II countries should consider prioritizing programs aimed at overall socio-economic improvement, including transport, communication, and sustained investment in girls’ education. Such programs can help further reduce maternal mortality.

Another factor that we found to be statistically significant in explaining reduction in MMR within the region was adolescent birth rate. Though data on adolescent birth rate was available over time for just seven countries in the MENA region, we determined that countries with a steep decline in MMR over the last 15 years witnessed a significant decline in adolescent birth rate. Although the contribution of declining adolescent birth rates to maternal mortality has been a subject of controversy, our analysis offers further guidance regarding possible direct (low adolescent maternal mortality) or indirect (higher education, greater empowerment, and decrease in fertility) effects on reducing maternal mortality [28]. We believe there is sufficient evidence for countries in all obstetric stages to progressively provide support to adolescents with evidence-based information and optimal services to enable them to make informed decisions about their reproductive health.

While previously published studies have focused on corroborating factors associated with maternal mortality reduction at an aggregate level, our analysis segregates countries according to obstetric stages to inform future policies and interventions. Although an impressive reduction in maternal mortality has been achieved in the MENA countries to date, they must persevere to further reduce maternal mortality, possibly to levels close to zero [29]. Some MENA countries have achieved single digit maternal mortality levels. However, these countries must advance to stop all preventable maternal deaths. Further exploration and investment is required to achieve optimal safety for the lives of future mothers.

Though this study brings together the best available data on maternal mortality and associated factors in the region, there are limitations. The data establishes association only and not causation. However, strong association, when warranted, may guide public health programs and policies to improve maternal health [30]. Though we collected data from multiple sources, data for many indicators were not available consistently over the years for all countries. The choice of indicators related to the three delays model pooled from the Millennium Development Goals, and the optimal approach taken by the authors could mean that there is relevant information that was not considered. There are no global databases for the number of ambulances in a country, for instance. Our choice to include the number of motor vehicles was the best fit for this situation, but we found this indicator to be very strongly correlated with the GDP. Lack of a good indicator to associate with the second delay leading to MMR should be considered a limitation of this study.

## Conclusion

Maternal Mortality Ratio in the MENA region has reduced significantly over the last 15 years. However, inequity prevails. Wealthier countries are doing better than poorer countries. While complex geopolitical factors, including conflicts, will need long-term strategic solutions, there are proximate factors that countries can concentrate on in the pursuit of saving the lives of mothers. Greater investment in human resources for health personnel, particularly for nurses and midwives, measures to improve adolescent sexual and reproductive health, and greater investments in achieving gender parity in education would be immediate steps in the right direction. It is important that countries learn from their more successful neighbors regarding what they got right while they were at preceding obstetric stages. They can then customize these findings to their local context and plan their future investments accordingly. We call for more robust data collection of evidence-based information by all countries to better track their progress towards zero preventable maternal deaths during pregnancy and childbirth. Individual countries, in consultation with such health agencies as the World Health Organization, United Nations Population Fund, United Nations Children’s Fund and others, should focus on collecting relevant information and tracking evidence-based indicators for further progress.

## Supplementary Information

**Additional file 1.**

## Data Availability

Datasets from the current study are available from the corresponding author on reasonable request.
