# Supplementary material for: Maternal mortality in the Middle East and North Africa region – how could countries move towards obstetric transition stage 5?
Source: BMC Pregnancy Childbirth. 2022 Jul 8;22:552. doi: 10.1186/s12884-022-04886-7 (PMC9264591; doi:10.1186/s12884-022-04886-7)
Supplement: Supplementary file 1 — Additional file 1. [file 12884_2022_4886_MOESM1_ESM.docx]

**Supplementary table 1:**  Association between selected indicators and GDP per capita (average values reported during 2011-2015)

| **Indicator** | **unit** | **GDP (US$) Per capita *** | |
| --- | --- | --- | --- |
|  |  | **Number of countries with** | **Pearson Correlation*** |
|  |  | **available data** | **(p-value)** |
| MENA COUNTRIES | Number | 23 |  |
|  |  |  |  |
| Maternal Mortality Ratio (MMR) | per 100,000 live births | 23 | **-0.90 (<.0001)** |
| Proportion of births attended by skilled health personnel | percentage | 13 | **0.69 (0.009)** |
| Contraceptive use among married women 15-49 years, any method | percentage | 12 | 0.32 (0.32) |
| Contraceptive use among married women 15-49 years, modern methods | percentage | 12 | 0.33 (0.29) |
| Adolescent birth rate | per 1,000 women | 9 | -0.66 (0.05) |
| Antenatal care coverage (At least one visit) | percentage | 14 | **0.56 (0.04)** |
| Antenatal care coverage (At least four visits) | percentage | 12 | **0.57 (0.05)** |
| Unmet need for family planning, total | percentage | 10 | -0.44 (0.20) |
| Unmet need for family planning, spacing | percentage | 10 | -0.27 (0.45) |
| Unmet need for family planning, limiting | percentage | 10 | -0.57 (0.09) |
| Gender Parity Index in primary level enrolment | Girls/boys ratio | 17 | **0.72 (0.001)** |
| Gender Parity Index in secondary level enrolment | Girls/boys ratio | 18 | **0.66 (0.003)** |
| Gender Parity Index in tertiary level enrolment | Girls/boys ratio | 18 | **0.71 (0.001)** |
| Share of women in wage employment in the non-agricultural sector | Women to men ratio | 9 | -0.46 (0.21) |
| Proportion of seats held by women in national parliament | Women to men ratio | 22 | -0.38 (0.08) |
| Nursing and midwifery personnel | per 10,000 population | 22 | **0.83 (<.0001)** |
| Medical doctors | per 10,000 population | 22 | **0.72 (0.0002)** |
| Motor vehicle ownership | per 1000 inhabitants | 23 | **0.85 (<.0001)** |
| Motorization rate 2014-15 | Per 1000 inhabitants | 21 | **0.75 (<.0001)** |

* Pearson correlation coefficient based on log-transformed values
